# Outbreak of *Salmonella* Heidelberg Infections Linked to a Single Poultry Producer — 13 States, 2012–2013

**Published:** 2013-07-12

**Authors:** Margaret Grinnell, Ginger Provo, Nicola Marsden-Haug, Kathleen A. Stigi, Emilio DeBess, Bonnie Kissler, Emily Crarey, Heather Tate, Jeshua Pringle, Julian Grass, Jason Folster, Ian Williams, Laura Gieraltowski, Alison S. Laufer

**Affiliations:** Alaska Dept of Health and Social Svcs; Washington State Dept of Health; Oregon Health Authority; Food Safety and Inspection Svc, US Dept of Agriculture; Center for Veterinary Medicine, Food and Drug Admin; Div of Foodborne, Waterborne, and Environmental Diseases, National Center for Emerging and Zoonotic Infectious Diseases; EIS Officer, CDC

In June 2012, the Oregon Health Authority and the Washington State Department of Health noted an increase in the number of *Salmonella enterica* serotype Heidelberg clinical isolates sharing an identical pulsed-field gel electrophoresis (PFGE) pattern. In 2004, this pattern had been linked to chicken from Foster Farms by the Washington State Department of Health; preliminary 2012 interviews with infected persons also indicated exposure to Foster Farms chicken. On August 2, 2012, CDC’s PulseNet* detected a cluster of 19 *Salmonella* Heidelberg clinical isolates matching the outbreak pattern. This report summarizes the investigation by CDC, state and local health departments, the U.S. Department of Agriculture’s Food Safety and Inspection Service (USDA-FSIS), and the Food and Drug Administration (FDA) and reinforces the importance of safe food handling to prevent illness. A total of 134 cases from 13 states were identified, including 33 patients who were hospitalized. This multifaceted investigation used standard epidemiologic and laboratory data along with patient shopper card purchase information, and PFGE data from the retail meat component of the National Antimicrobial Resistance Monitoring System (NARMS)†, a relatively novel tool in outbreak investigation, to link the outbreak strain to chicken from Foster Farms.

## Epidemiologic Investigation

**FIGURE 1:**
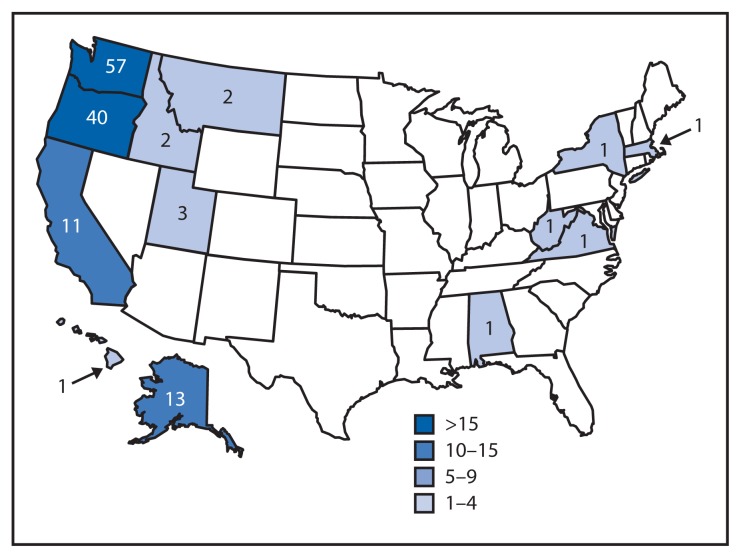
Number of persons (N = 134) infected with the outbreak strain of *Salmonella* Heidelberg, by state — United States 2012–2013

A total of 134 persons infected with the *Salmonella* Heidelberg outbreak strain§ with illness onset on or after June 1, 2012, were identified in 13 states (Figure 1). Median patient age was 22 years (range: <1 to 94 years); 73 (55%) of 132 patients with data available were female. Illness onset ranged from June 4, 2012, to April 16, 2013. Thirty-three (31%) of 105 patients with known outcomes were hospitalized; no deaths were reported. The majority of cases were reported from four states in the Pacific Northwest: Washington, 57 cases; Oregon, 40; Alaska, 13; and California, 11 (Figure 1). This outbreak appears to have ended, based on the calculated 5-year baseline of the expected number of cases reported per week.

Initial state-based interviews found that chicken was commonly consumed by the persons with infections. A structured questionnaire was developed to collect detailed information on chicken and other exposures noted during initial interviews, and exposures commonly linked to *Salmonella* Heidelberg, such as eggs. Of 70 patients who responded, 55 (79%) reported consuming chicken in the week before illness onset, a percentage significantly higher (p=0.01) than the 64.9% reported in the 2006–2007 Foodborne Diseases Active Surveillance Network (FoodNet) Population Survey of healthy persons.¶ In addition, eight patients reported that chicken had been prepared in the home, but either had not been consumed or consumption was not specified. In total, 36 (71%) of 51 patients who had brand information available reported exposure either to Foster Farms chicken (27 patients) or to another brand likely produced by Foster Farms (although packaging information was unavailable) (nine patients). Other exposures of interest (e.g., eggs) were reported in significantly lower proportions by patients than by respondents to the FoodNet survey.

NARMS, a collaboration of CDC, FDA’s Center for Veterinary Medicine, USDA, and participating state public health laboratories, monitors the prevalence and trends of antimicrobial resistance among enteric bacteria from humans, raw unprocessed retail meats, and food animals. Of 14 clinical isolates from this outbreak tested for antimicrobial susceptibility by NARMS, 12 were susceptible to all antimicrobials tested, and two were resistant to amoxicillin/clavulanic acid, ampicillin, cefoxitin, ceftiofur, and ceftriaxone. The two patients with resistant isolates both were aged <12 months and required hospitalization; for both patients, exposure to Foster Farms chicken was reported. Resistance was mediated by the presence of an IncI1 plasmid carrying a *bla*_CMY-2_ gene. Plasmids are mobile genetic elements that can be gained or lost relatively easily, which might explain the variable resistance profiles. Resistance to third-generation cephalosporins (e.g., ceftriaxone) is clinically important because extended-spectrum cephalosporins are commonly used for treatment of severe salmonellosis in children (1).

## Product Testing and Traceback Investigation

Oregon and Washington worked with USDA-FSIS to conduct a traceback investigation using shopper card records from nine patients. Records indicated all nine patients purchased Foster Farms chicken before illness onset. Four intact (i.e., unopened) chicken samples from three Washington patients’ homes were tested for *Salmonella*; all yielded the outbreak strain and were traced back to two Foster Farms slaughter establishments. Three were susceptible to all antimicrobials tested; one was resistant to gentamicin, streptomycin, and sulfisoxazole. As part of this investigation, USDA-FSIS sent an incident investigation team to one Foster Farms slaughter establishment; the results of that investigation have not yet been finalized.

## NARMS Retail Samples

The NARMS retail meat surveillance program isolated the outbreak strain from Foster Farms retail chicken samples purchased in Washington and Oregon in October 2012. Using PFGE data provided by the Center for Veterinary Medicine, the association between Foster Farms chicken and the outbreak strain was evaluated. From 2002 to 2011, *Salmonella* was isolated from 1,503 (13%) of 11,417 retail chicken samples tested by NARMS, of which 233 (16%) were serotype Heidelberg. Among these, 48 (21%) matched the outbreak strain, of which 47 (98%) were Foster Farms retail chicken isolates. Stratification by brand showed that 47 (52%) of 90 NARMS Foster Farms chicken isolates matched the outbreak strain, compared with one (0.7%) of 143 isolates not from Foster Farms (p<0.001).

## Public Health Response

The two state health departments and CDC issued Internet announcements and news releases regarding the outbreak investigation, indicating that Foster Farms chicken was the most likely source of the outbreak, that antimicrobial testing revealed most of the isolates were susceptible to all antimicrobial agents tested, and reminding the public of the importance of safe handling of raw poultry.

What is already known on this topic?

Poultry is the commodity most frequently associated with *Salmonella* outbreaks. *Salmonella* is the most common bacterial cause of foodborne illness in the United States, and is estimated to cause approximately 1 million illnesses annually.

What is added by this report?

An outbreak of 134 *Salmonella* Heidelberg cases in the Pacific Northwest was linked to chicken consumption. Information from National Antimicrobial Resistance Monitoring System (NARMS) retail meat surveillance and shopper card records helped link the outbreak strain with Foster Farms.

What are the implications for public health practice?

The historical significance of this pattern in the Pacific Northwest suggests the need for ongoing surveillance and intervention to prevent additional illnesses. Shopper card records and NARMS retail meats surveillance can provide brand information crucial to investigations of outbreaks linked to commonly consumed foods. Because it is not unusual that raw poultry from any producer has *Salmonella,* it is important to continue to remind consumers of the need for safe raw poultry handling practices to help the public protect themselves and others from foodborne illness.

### Editorial Note

Epidemiologic data, traceback investigations, and product testing support the conclusion that Foster Farms chicken was the likely source of this outbreak. Shopper card records collected from patients provided specific brand information for chicken, a commonly consumed food product, and were critical to linking this outbreak to a single chicken producer. The NARMS retail meat surveillance program not only isolated the outbreak strain from Foster Farms retail chicken samples purchased in Oregon and Washington during the current outbreak, but also demonstrated that 98% of historic isolates matching the outbreak strain were from Foster Farms retail chicken samples. One limitation to these findings is that they might not reflect all establishments that produce Foster Farms chicken or all brands of chicken produced by each establishment.

**FIGURE 2:**
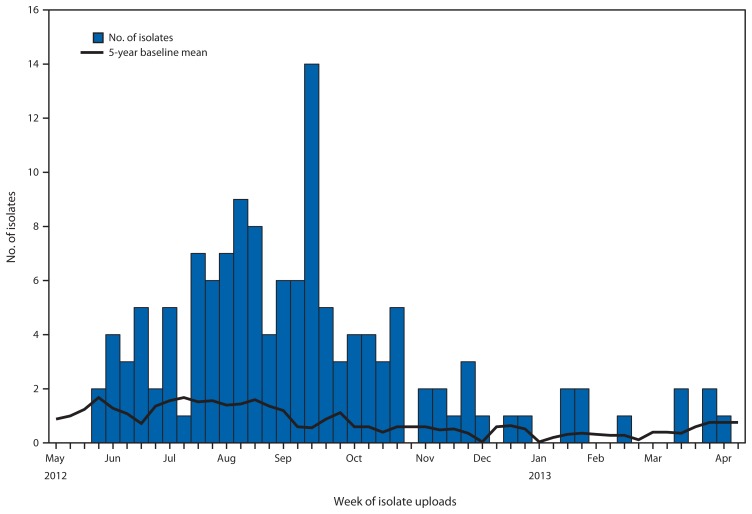
Number of clinical isolates matching the *Salmonella* Heidelberg outbreak strain and 5-year baseline mean number of cases with the same strain, by week of uploads — PulseNet,^*^ United States, 2012–2013 ^*^ Additional information available at http://www.cdc.gov/pulsenet.

PulseNet data collected before this outbreak indicate that four to eight human isolates of this Heidelberg pattern typically are uploaded each month from June to November (Figure 2). During this outbreak, an average of 12 human isolates matching the outbreak strain was uploaded each month. The proportion of *Salmonella* Heidelberg human isolates uploaded to PulseNet with this PFGE pattern also has been increasing: from 3.5% to 5.7% of all *Salmonella* Heidelberg uploads per year during 2004–2008, to 3.7% to 13.7% during 2009–2012.

**FIGURE 3:**
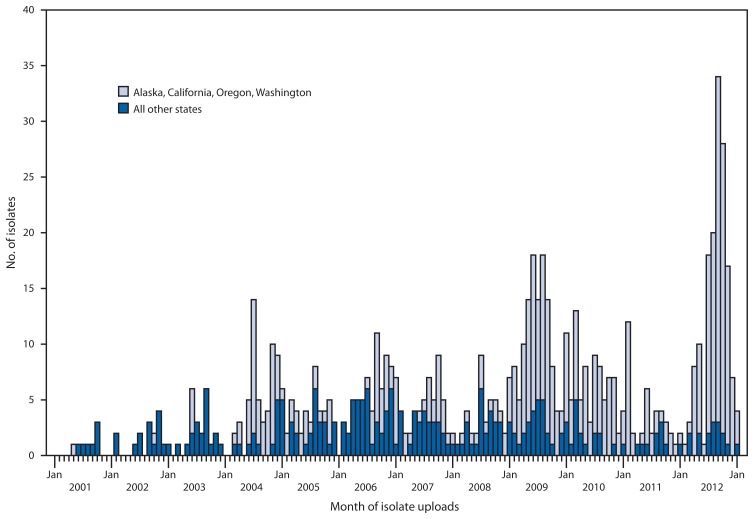
Comparison of the number of clinical isolates matching the *Salmonella* Heidelberg outbreak strain from Alaska, California, Oregon, and Washington with the number from all other states, by month of uploads — PulseNet,^*^ United States, 2001–2012 ^*^ Additional information available at http://www.cdc.gov/pulsenet.

Historically, reports of this pattern to PulseNet come from the Pacific Northwest region of the United States. Foster Farms chicken was previously linked to illness in a 2004 investigation by Washington and USDA-FSIS (Kathryn MacDonald, Washington State Department of Health, personal communication, 2012). USDA-FSIS conducted comprehensive food safety assessments in 2004 and 2009. Following the 2004 assessment, USDA-FSIS issued a Notice of Intended Enforcement to Foster Farms, after which uploads to PulseNet of the outbreak strain decreased, followed by an increase in 2009 (Figure 3). From 2001 to 2012, *Salmonella* was isolated from 3,094 (4%) of 83,743 raw meat and poultry samples collected for testing by USDA-FSIS from establishments in the western United States. Among these, 264 (9%) were serotype Heidelberg, 45 (17%) of which matched the outbreak strain. The historical significance of this pattern in the Pacific Northwest suggests the need for ongoing surveillance and intervention to prevent additional illnesses.

*Salmonella* Heidelberg is frequently isolated from retail meats and predominantly from poultry products; in 2010, 38% of *Salmonella* Heidelberg strains isolated from retail chicken breasts were resistant to at least one antimicrobial class (2). Raw poultry in general can have *Salmonella*, and *Salmonella* is not considered a bacterial contaminant in raw poultry from a regulatory perspective. However, poultry is the commodity most often associated with *Salmonella* outbreaks (3). Therefore, consumers should follow safe handling instructions to help protect themselves and others from foodborne illness. USDA-FSIS set stricter pathogen-reduction performance standards for *Salmonella* contamination in young chicken and turkey carcasses at slaughter facilities, effective July 2011. In December 2012, USDA-FSIS announced that all establishments producing not-ready-to-eat ground or comminuted poultry products, including Foster Farms, will be required to reassess their Hazard Analysis and Critical Control Points plans, in response to recent turkey-associated outbreaks of salmonellosis (4,5).

This outbreak illustrates the importance of a multifaceted outbreak investigation, and particularly the value of incorporating historical PulseNet and NARMS data with information from patient interviews, shopper card records, and product samples from patients’ homes. NARMS retail meat surveillance data is a relatively novel and useful tool to help link an outbreak strain with a particular brand and should be considered in future foodborne disease outbreak investigations.

## Footnotes

* Additional information available at http://www.cdc.gov/pulsenet.

† Additional information available at http://www.fda.gov/animalveterinary/safetyhealth/antimicrobialresistance/nationalantimicrobialresistancemonitoringsystem/default.htm.

§ *Xba*I pattern JF6X01.0122.

¶ Additional information available at http://www.cdc.gov/foodnet/studies/population-surveys.html.
